# Single-Center Comparative Evaluation of Freehand Transperineal and Transrectal Prostate Biopsy Techniques Performed Under Local Anesthesia

**DOI:** 10.3390/diagnostics15151929

**Published:** 2025-07-31

**Authors:** Laurian Ștefan Maxim, Ruxandra Maria Rotaru, Camelia Cornelia Scârneciu, Marius Alexandru Moga, Florin Lucian Petrică Sabou, Anda Catica Hogea, Raul Dumitru Gherasim, Alexandru Ghicavîi, Razvan-Dragos Mulțescu, Mihail-Alexandru Badea, Bogdan Ovidiu Feciche, Ioan Scârneciu

**Affiliations:** 1 Emergency Clinical County Hospital Brasov, 500365 Brașov, Romania; maxim.laurian.stefan@unitbv.ro (L.Ș.M.); ruxandra.manisor@unitbv.ro (R.M.R.);; 2 Faculty of Medicine, Transilvania University of Brasov, 500019 Brașov, Romania; 3 Department of Urology, Clinical County Hospital Mures, 540136 Târgu Mures, Romania; 4 Department of Urology, “Saint John” Clinical Emergency Hospital, 042122 Bucharest, Romania; 5 Natural Skin Târgu Mures, 540357 Mureș, Romania; 6 Department of Urology, Emergency County Hospital Oradea, 410169 Oradea, Romania

**Keywords:** prostate biopsy, transperineal, transrectal, prostate cancer, local anesthesia, prostate cancer, detection rate

## Abstract

**Background:** To diagnose prostate cancer, a prostate biopsy is required. Two methods are commonly used for biopsy: transrectal and transperineal. The transperineal approach, particularly the “freehand” technique under local anesthesia, offers better access to the anterior prostate, lower infection risk, and higher detection rates. **Methods:** This retrospective study examined the records of 1674 patients who underwent ultrasound-guided prostate biopsies between 2015 and 2022. Of these, 1161 patients had transperineal biopsy using the “freehand” method, and 513 had transrectal biopsy. All the biopsies were carried out under local anesthesia, with a combined systematic and targeted approach for patients with MRI-identified lesions. **Results:** This study demonstrates that the transperineal biopsy approach significantly increased the detection rate of clinically significant prostate cancer compared with the transrectal method, with detection rates of 65.7% and 59.4%, respectively. Notably, the transperineal technique also achieved superior detection of anteriorly located tumors (94.1% vs. 43.1%), supporting its use as the preferred biopsy strategy, particularly in anatomically challenging regions. Moreover, patients who underwent transperineal biopsy demonstrated more favorable diagnostic outcomes, characterized by a higher detection rate for clinically significant cancers and a reduced incidence of clinically insignificant cases. The transperineal method outperformed the transrectal approach, especially among younger patients and those presenting with lower PSA values. These results highlight the diagnostic superiority and broader clinical applicability of the transperineal biopsy technique across various patient subgroups.

## 1. Introduction

Prostate biopsy continues to be a routine and essential procedure in the diagnosis of prostate cancer. It remains the definitive method for diagnosing prostate cancer, essential for confirming malignancy and guiding treatment decisions [1]. In 2022, there were approximately 1.47 million new cases of prostate cancer globally [2,3], reflecting its high prevalence and the critical need for effective diagnostic tools, underscoring the significant prevalence of suspected prostate cancer cases and the ongoing demand for precise and effective diagnostic tools to support clinical decision-making and improve patient outcomes.

Despite significant advances in the understanding of prostate cancer biology and associated risk factors, the disease remains the most commonly diagnosed malignancy among men in Europe. According to GLOBOCAN 2022 estimates, prostate cancer remains the second most commonly diagnosed cancer among men worldwide, with a global incidence rate of 29.4 per 100,000 [4]. Globally, incidence rates vary considerably, with the highest reported in developed regions such as Western Europe, North America, and Australia. This variation is likely driven by disparities in life expectancy, healthcare access, and the widespread implementation of prostate-specific antigen (PSA) screening, as well as variations in cancer registry reporting practices, all of which contribute to regional differences in prostate cancer incidence and diagnosis. This is probably because people in these regions live longer, have better access to healthcare, and use PSA testing more often. Still, prostate cancer remains a challenge because it can be hard to tell which tumors are slow-growing and which are more dangerous. Notably, prostate cancer has a significantly higher incidence compared with lung cancer, which ranks second.

The exact causes of prostate cancer are still not fully understood, and there is a need for more research, especially in large population studies, to improve prevention and better understand how the disease develops. Established risk factors for prostate cancer include older age, a positive family history, and ethnicity, with men of African descent having higher incidence rates and more aggressive disease [5]. These disparities suggest potential genetic, environmental, and socioeconomic determinants contributing to the higher disease burden in this population [6].

Prostate biopsy is typically indicated when a digital rectal examination (DRE) raises suspicion or when prostate-specific antigen (PSA) levels remain persistently elevated, as these factors are strongly associated with the potential presence of prostate cancer. However, an isolated elevation in PSA does not necessarily warrant immediate biopsy, as PSA is a non-specific marker and may be influenced by various benign conditions. These include recent ejaculation, lower urinary tract infections, prostatitis, or recent urological procedures, all of which can transiently elevate PSA levels [7]. As such, clinical context and repeat testing are essential for appropriate decision-making prior to proceeding with invasive diagnostic evaluation. Therefore, in cases of borderline or isolated PSA elevation, it is recommended to repeat the measurement after several weeks to rule out transient increases before proceeding with invasive diagnostic procedures [8,9].

With regard to biopsy techniques, two main approaches are used to detect prostate cancer: the transrectal and transperineal methods. These techniques differ in several aspects, including the access route, puncture site, type of endorectal transducer used, patient positioning, cancer detection rates, and procedure-specific complications. In a transrectal biopsy, the needle is inserted through the anterior rectal wall, guided by transrectal ultrasound. In contrast, a transperineal biopsy involves advancing the needle through the perineum, also guided by transrectal ultrasound, typically using a biplane transducer [8].

Transperineal biopsy techniques have evolved significantly over time, transitioning from the use of a brachytherapy-style perineal template grid typically performed under general anesthesia, to a more simplified, cognitively guided “freehand” technique conducted under local anesthesia. Both methods are well established and clinically validated; however, their adoption and routine use may vary depending on the resources, infrastructure, and procedural preferences within different healthcare systems [9]. The shift away from the perineal template has expanded possibilities for performing the procedure in an outpatient setting using local anesthesia, eliminating the need for additional equipment. The transperineal prostate biopsy technique offers a lower risk of sepsis and improved sampling of anterior and apical regions, making it a safer and more accurate alternative to the transrectal approach, particularly in the context of rising antimicrobial resistance [10,11,12,13] Additionally, the risk of seeing needle advancement through the perineum is nearly nonexistent [13].

Anesthesia is a critical component of the biopsy procedure. Although many patients can tolerate the procedure, discomfort may limit the procedure and discourage patients from consenting to a rebiopsy if needed. Periprostatic anesthesia has been proven effective in reducing pain during the procedure [14,15]. Local anesthesia is well tolerated in the context of transperineal prostate biopsy and has been shown to be feasible in routine clinical practice. Comparative analyses indicate no substantial differences between the transperineal and transrectal approaches with respect to patient tolerability or procedural duration [16,17].

Collection of biopsy fragments via the “freehand” technique under local anesthesia has transformed an initially complicated procedure that required general anesthesia and a grid for collecting fragments. Recent studies confirm that transperineal prostate biopsy using the freehand technique under local anesthesia has transformed a previously complex procedure into a feasible outpatient method. While it presents a steeper learning curve compared to the transrectal approach, it offers improved patient comfort and reduced infection risks [18].

### Study Objective

The objective of this study was to compare the diagnostic performance of transperineal and transrectal ultrasound-guided prostate biopsy techniques, focusing on the detection of clinically significant prostate cancer, especially in anterior prostate regions. The study also aimed to assess the effectiveness of the freehand transperineal approach under local anesthesia across different patient subgroups.

## 2. Materials and Methods

### 2.1. Study Design

Between 2015 and 2022, a total of 1674 patients who met the inclusion criteria were retrospectively evaluated at the Urology Clinic of Brașov County Emergency Hospital. Of these, 1161 patients underwent cognitively guided, ultrasound-assisted transperineal prostate biopsy using the freehand technique, whereas 513 patients underwent ultrasound-guided transrectal biopsy.

Inclusion criteria for the study were as follows: serum PSA level >4 ng/mL, clinical suspicion of malignancy based on digital rectal examination (DRE), or imaging findings suggestive of prostate cancer on multiparametric MRI or transrectal ultrasound with elastography. Patients with PSA levels exceeding 1000 ng/mL were excluded from the analysis to minimize potential bias related to advanced or metastatic disease presentation.

Demographic, clinical, and pathological data were comprehensively collected through a retrospective analysis of patient medical records. The variables extracted included patient age, body mass index (BMI), history of diabetes mellitus, use of anticoagulant therapy, serum prostate-specific antigen (PSA) levels, prostate volume, the total number of biopsy cores obtained, and pertinent imaging findings, including those from multiparametric magnetic resonance imaging (MRI) and transrectal ultrasound. Prostate volume was assessed via transrectal ultrasonography (TRUS) and calculated using the standard ellipsoid formula: width × length × height × 0.52. This approach ensured consistent volumetric estimation across the study cohort.

All patients provided written consent after being fully informed about the details of each procedure and the potential complications. All study protocols were conducted in strict adherence to the Declaration of Helsinki, ensuring ethical standards were met throughout the research. Patient data were handled and processed in full compliance with applicable data protection and privacy regulations, including relevant local and international laws.

The protocol for the collection of prostatic fragments in the case of transperineal and transrectal biopsy was in most patients a combined collection procedure between systematic biopsy and targeted biopsy in case patients had prostatic lesions identified by MRI.

All biopsy specimens were appropriately labeled, meticulously processed, and independently assessed by a team of experienced pathologists, following the established guidelines of the International Society of Urological Pathology (ISUP). For consistency and standardization, all specimens were reviewed using contemporary classification systems. Clinically significant prostate cancer (csPCa) was defined as Gleason grade group (GG) ≥ 2, in accordance with current histopathological standards, ensuring that only clinically relevant cases were classified as csPCa.

Patient data were recorded and managed using Microsoft Excel (Microsoft Corp., Redmond, WA, USA). Statistical analysis was conducted using IBM SPSS Statistics for Windows, version 23.0 (IBM Corp., Armonk, NY, USA) and the MatchIt package in R, version 4.5.1. Descriptive statistics were applied to summarize demographic and clinical data, and group comparisons were made using appropriate statistical tests, with significance set at *p* < 0.05.

### 2.2. Biopsy Protocol for Transperineal Prostate Biopsy

All procedures were performed under aseptic conditions within a dedicated operating theater. Transperineal prostate biopsies were conducted utilizing a BK Medical ultrasound system (GE Healthcare - Peabody, MA, USA), equipped with a biplane endorectal transducer (Model 8848), enabling real-time guidance and anatomical visualization. Biopsy was performed using a Magnum automatic biopsy device with a 22 mm cutting length, employing 16- and 18-gauge Tru-Cut biopsy needles to ensure adequate core sampling.

In accordance with institutional protocols for infection prophylaxis, a single pre-procedural dose of antibiotics was administered to all patients. Temporary urinary catheterization was selectively performed in cases where anteriorly located lesions were identified on imaging, to facilitate access and minimize discomfort; in such cases, catheterization was maintained only for the minimal necessary duration.

### 2.3. Local Anesthesia Protocol

In the transperineal group, local anesthesia was administered at the level of the anterior (urogenital) perineal triangle, encompassing the base of the scrotum and penis.

The initial site of infiltration was located approximately 4–5 cm superior to the anal verge, along the midline perineal raphe. At this level, 10 mL of 1% lidocaine was injected subcutaneously to achieve surface anesthesia of the skin and underlying subcutaneous tissues.

The second infiltration targeted the periprostatic space. Under real-time transperineal ultrasound guidance, an additional 10 mL of 1% lidocaine was administered using a spinal-type puncture needle. This approach facilitated effective pudendal nerve blockade, ensuring adequate analgesia for deeper perineal structures during the procedure.

The “freehand” technique, performed through a single incision, requires a higher learning curve than transrectal biopsy, and the learning curve is longer compared to other types of transperineal biopsy that use systems by which the needle is directed to the prostate area through a guidance system mounted on the transducer. 

All patients were admitted for observation following the procedure and discharged after one day.

In the transrectal (TR) group, a periprostatic nerve block was performed transrectally using 10 mLof 1% lidocaine bilaterally.

For the prostate biopsies, no pain assessment scale was used; all procedures were successfully performed under local anesthesia without the need for intravenous anesthesia, ensuring patient comfort while preserving procedural efficiency and safety.

## 3. Results

### 3.1. Cohort Study Characteristics

The characteristics of the cohort are outlined in Table 1.

Those who underwent a transperineal biopsy were aged between 69.87 ± 7.71 and those who had a transrectal biopsy were aged between 72.19 ± 7.81. The Student’s *t*-test indicated a significant difference in age between the two groups, with patients in the TPPBx group being notably older than those in Group 1, who underwent transrectal biopsy. From the point of view of the PSA value, patients in group I (TPPBx) had an average PSA of 53.8437 ± 128.29 ng/mL, compared with those in group II who had an average PSA of 72.7547 ± 162.23 ng/mL (TRPBx). The Mann–Whitney test revealed a significant difference, with patients in the TRPBx group showing notably higher values compared with those in Group I.

Prostate volume was comparable between the two groups, with statistical analysis using the Student’s *t*-test showing no significant differences between them. These results suggest that prostate volume did not differ substantially between the groups in this study. From the point of view of the number of fragments collected by the two procedures, there was a significant difference highlighted based on the Student’s test, transperineal biopsied patients have a higher number of fragments collected compared to those biopsied transrectally. Comorbidities such as obesity, diabetes, and anticoagulant use were more common in those with TPPBx compared with those with TRPBx (*p* = 0.915, *p* = 0.003, *p* = 0.024). Patients identified with different degrees of obesity by TPPBx and TRPBx did not show statistical significance. In patients who used anticoagulants, which were discontinued before the biopsy, statistically significantly more patients who had used oral anticoagulants were identified in group I. As with the use of anticoagulants, there was a statistically significant difference in patients who had diabetes in group I versus those who were included in group II.

To minimize selection bias and achieve comparable groups, we performed propensity score matching (PSM) using the MatchIt package in R (version 4.5.1). A 1:1 nearest-neighbor matching without replacement was applied, with the treatment variable being the biopsy approach (transperineal vs. transrectal). The following covariates were included in the model for estimating the propensity scores: age (mean ± standard deviation, median), PSA level (mean ± SD, median), digital rectal examination (yes/no), prostate volume (mean ± SD, median), number of biopsy cores (mean ± SD, median), obesity (yes/no), anticoagulant use (yes/no), diabetes (yes/no), MRI (yes/no), ultrasound with elastography (yes/no), and prior prostate biopsies (yes/no). After matching, 513 patients were retained in each group (group I transperineal and group II transrectal). Normality tests (Shapiro–Wilk) performed on the matched sample showed that none of the continuous variables met the assumption of normal distribution in either group; therefore, non-parametric tests (Mann–Whitney U) were used for continuous variables, while categorical variables were analyzed with the Chi-square test for group comparisons after PSM. Even after the application of propensity score matching (PSM) techniques, the initial findings continue to hold, demonstrating their robustness and internal validity.

### 3.2. Incidence of Prostate Cancer

The mean age of patients diagnosed with prostate cancer through biopsy was 70.74 years, with ages ranging from 46 to 93 years. Statistical analysis revealed no significant difference in the mean age between patients with histologically confirmed prostate cancer and those without malignancy (*p* > 0.05). These results suggest that age, by itself, was not a significant factor in the detection of prostate cancer within this cohort. Both patient groups demonstrated a comparable mean age of approximately 70 years, suggesting that, in the context of this study, age was not a substantial predictor of prostate cancer diagnosis (Table 2).

The Student’s *t*-test showed a statistically significant difference in mean PSA levels between patients diagnosed with prostate cancer and those without malignancy. The cancer group had a much higher mean PSA level of 80.73 ng/mL, compared with 19.94 ng/mL in the non-cancer group (*p* < 0.05). This highlights the strong link between elevated PSA levels and a higher likelihood of prostate cancer, supporting the use of PSA as a key biomarker in diagnosing and assessing the risk of prostate cancer.

Furthermore, the number of transperineal biopsies performed was significantly greater among patients with confirmed prostate cancer (*n* = 788) than in those without malignancy (*n* = 373), suggesting increased diagnostic yield or biopsy intensity in this subgroup. A similar pattern was observed in the transrectal biopsy cohort, where a higher number of procedures were recorded in patients diagnosed with prostate cancer compared to those with benign histology (*n* = 305 vs. *n* = 208, respectively).

No statistically significant difference in prostate volume was identified between the two groups. The mean prostate volume was 63.6 cm^3^ in the cancer group and 65.8 cm^3^ in the non-cancer group (*p* > 0.05), suggesting that prostate volume was not a significant factor in differentiating between malignant and non-malignant cases within this cohort. Other clinical factors, beyond prostate volume, may be necessary to improve the accuracy of prostate cancer diagnosis.

### 3.3. Overall Efficacy in Detecting Clinically Significant and Clinically Insignificant Prostate Cancer According to Biopsy Approach

Among the 1093 patients with a positive transperineal or transrectal biopsy indicating prostate cancer, 788 (67.8%) were diagnosed via transperineal biopsy and 305 (59.4%) through transrectal biopsy. Among patients without premalignant lesions, the transperineal biopsy approach demonstrated a significantly higher overall detection rate for prostate cancer compared with the transrectal technique (65.7% vs. 57.3%; *p* < 0.05). Additionally, the transperineal biopsy approach identified a significantly higher proportion of clinically insignificant tumors (8.1% vs. 4.3%; *p* < 0.05) compared with the transrectal method. While the transperineal approach also detected a greater proportion of clinically significant prostate cancers (57.6% vs. 53.0%), this difference did not reach statistical significance (*p* > 0.05) (Table 3).

### 3.4. Prostate Cancer Detection Based on Biopsy Approach and Gleason Score

Gleason score 8 was most common in 262 patients in the group of TP biopsy, followed by Gleason score 7 (4-3) in 157 patients. In the transrectal biopsy group, the most frequently observed Gleason score was 8, followed by Gleason score 9 in 63 patients. In the TRPBx group, more patients were assigned in aggressive risk groups compared to those in the TPPBx group (Table 4).

### 3.5. Detection Rate Within the Anterior Prostate Zone

A total of 102 patients with suspicious lesions in the anterior zone of the prostate, identified through MRI, underwent transperineal prostate biopsy. The cancer detection rate in this group was 94.1% (96 out of 102 patients). Most of the detected cancers were acinar adenocarcinomas, found in 93.1% of cases (95 patients).

The detection rate for anterior prostate cancer was significantly higher with the transperineal biopsy compared with the transrectal method (94.1% vs. 56.9%). This suggests that the transperineal approach is more effective in detecting tumors in the anterior prostate, which are often missed with the transrectal biopsy.

### 3.6. Classification of Prostate Cancer Patients into Risk Groups Based on Age

Patients not diagnosed with prostate cancer were statistically more likely to be in the 61–75-year age group and underwent transrectal prostate biopsy (TRPBx) (45.6% vs. 36.4%, *p* < 0.05). Additionally, patients over 75 years of age were more likely to be in the group without prostate cancer and underwent transrectal biopsy (40.4% vs. 26.5%, *p* < 0.05). Among patients in the 61–75-year age group classified as low-risk, more were identified using the transperineal approach (13.1% vs. 8.8%, *p* > 0.05) (Table 5).

Among intermediate-risk patients, a higher proportion of individuals aged 61–75 years were diagnosed with prostate cancer using the transperineal biopsy compared with the transrectal method (26.4% vs. 19.4%). However, this difference was not statistically significant (*p* > 0.05), suggesting that age did not significantly affect the diagnostic advantage of the transperineal approach in this group. In contrast, a significant difference was observed in patients over 75, where a higher proportion of prostate cancer cases wasdetected using the transperineal technique compared with the transrectal method (30.4% vs. 18.0%, *p* < 0.05).

In the high-risk group, transrectal biopsy identified more patients in the 50–60- and 61–75-year age groups than transperineal biopsy (30.8% vs. 23.2% and 26.2% vs. 24.1%; *p* > 0.05).

### 3.7. Risk Stratification of Prostate Cancer Based on Prostate-Specific Antigen (PSA) Levels

Prostate cancer detection rates were significantly higher in patients who underwent transperineal biopsy compared to those who underwent transrectal biopsy, regardless of pre-biopsy PSA levels (*p* < 0.05). Notably, in patients with a Gleason grade group (GG) ≥ 3, the transperineal biopsy technique demonstrated superior detection of clinically significant cancers, particularly in individuals with PSA levels between 10 and 20 ng/mL (*p* < 0.05). These results highlight the transperineal approach’s enhanced sensitivity in identifying clinically significant prostate cancer, particularly in the intermediate PSA range, where detection challenges are most prevalent (Table 6).

However, no statistically significant differences in prostate cancer detection rates were observed between the transperineal and transrectal biopsy techniques in patients with PSA levels either below 10 ng/mL or exceeding 20 ng/mL. Similarly, for individuals with PSA >20 ng/mL, no significant differences were noted between the transperineal and transrectal approaches when stratified by risk groups with GG ≥2 or ≥3. These findings suggest that the transperineal approach offers the greatest diagnostic advantage in patients with intermediate PSA values and higher-grade disease.

## 4. Discussion

Prostate cancer incidence and biopsy rates are expected to rise significantly by 2040, primarily due to increased screening uptake and population aging [19].

Transperineal biopsy techniques have evolved from the use of a perineal brachytherapy template grid, requiring general anesthesia, to the freehand technique, which can be performed under local anesthesia. While both methods are well established, their application varies significantly across different healthcare systems [11]. The use of a perineal template has constrained the transperineal biopsy procedure, as it necessitates general anesthesia, thereby increasing associated costs and surgical risks. 

In the current study, the transperineal prostate biopsy procedures were performed by a single experienced urologic surgeon to ensure consistency and minimize operator-related variability. The transrectal prostate biopsies were performed by urologic surgeons with established experience in this procedure. For the transperineal approach, a learning curve of approximately 40 cases was required to achieve procedural proficiency.

Advancing age is a key risk factor for prostate cancer, with incidence rates increasing significantly after the age of 55. The occurrence of prostate cancer in individuals under 50 is very rare, with rates reported as low as 0.1% [19]. Consistent with these epidemiological trends, our study identified only 4 cases (0.2%) of prostate cancer among 1674 patients under the age of 50 who presented with clinical suspicion of the disease. These findings reinforce the strong correlation between age and prostate cancer risk and further highlight the rarity of diagnosis in younger populations.

The widespread utilization of prostate-specific antigen (PSA) testing has fundamentally altered the landscape of prostate cancer detection, contributing to an increased frequency of diagnoses at earlier, localized stages, particularly among younger individuals. This shift has led to improved life expectancy for patients diagnosed with prostate cancer, particularly when treatment is initiated during the curative stage. However, before advocating for PSA screening, it is critical to discuss with patients the potential for identifying clinically indolent prostate cancer, which raises concerns about the risks of overdiagnosis and overtreatment. In the present study, patients undergoing transrectal biopsy exhibited significantly higher mean PSA levels compared with those undergoing transperineal biopsy (72.75 ± 162.23 ng/mL vs. 53.84 ± 128.29 ng/mL). Furthermore, transperineal biopsy patients were diagnosed at lower PSA levels and at earlier stages of tumor development.

The transperineal biopsy approach demonstrated an overall prostate cancer detection rate of 65.7%, with malignancy confirmed in 763 patients. Among the 788 patients diagnosed with prostate cancer, 25 cases (2.1%) presented with lesions suggestive of precursor stages of malignancy.

In comparison, within the cohort that underwent transrectal biopsy, the overall detection rate, including lesions potentially indicative of precursor pathology, was 59.4%. Based on Gleason score classification, definitive prostate cancer was diagnosed in 294 patients (57.3%) in the transrectal group. A statistically significant difference in detection rates was observed between the two biopsy techniques, with the transperineal approach demonstrating superior diagnostic performance (67.8% vs. 59.4%; *p* < 0.05). These findings support the increased sensitivity of the transperineal route, particularly in capturing both early-stage and clinically significant disease. Notably, studies by Gorin et al. and Neale et al. reported significantly higher detection rates of 83.2% and 84%, respectively. These studies were among the few to include all patients with at least one targeted biopsy, where lesions were previously identified on pre-biopsy MRI [20]. Tewes et al. reported a substantial difference in prostate cancer detection rates between the two biopsy techniques. In their study, the transrectal biopsy group demonstrated a detection rate of 39%, whereas the transperineal biopsy group achieved a significantly higher detection rate of 75%, highlighting the superior diagnostic yield of the transperineal approach [21]. However, in the meta-analysis by Xue et al. [22] and Xiang et al. [23], they observed no statistically significant differences in prostate cancer detection rates between the transrectal and transperineal biopsy methods. Both techniques displayed comparable diagnostic accuracy in identifying prostate cancer. Jiang et al. [24] conducted a study demonstrating that the transrectal biopsy approach yielded higher prostate cancer detection rates than the transperineal technique in patients with PSA levels ranging from 0.1 to 100 ng/mL (80.8% vs. 69.1%, respectively; *p* = 0.04).

Lesions located in the anterior zone of the prostate are inherently difficult to palpate and are frequently under-sampled or missed entirely during transrectal biopsy, primarily due to anatomical constraints and limited access to this region. These limitations underscore the need for alternative biopsy techniques that can more effectively target anterior lesions [25]. Furthermore, tumors in this region are more difficult to detect using MRI imaging [26]. To reduce the need for multiple transrectal biopsies, which are often impeded by limited access to the anterior prostate, and to prevent unnecessary systematic transperineal biopsies in patients with a high clinical suspicion of prostate cancer, it is recommended to incorporate pre-biopsy multiparametric MRI. This imaging technique enhances the identification of lesions in the anterior prostate, allowing more targeted and precise biopsy procedures, thereby improving diagnostic accuracy and minimizing procedural complications [27]. Tumors identified during follow-up prostate biopsies are most commonly located in the anterior part of the prostate [28,29,30], an area that is challenging to access via transrectal prostate biopsy but is more readily accessible through the transperineal approach [31,32]. The detection rate for prostate cancer in anterior prostate lesions was significantly higher with the transperineal biopsy technique compared with the transrectal approach, with rates of 94.1% and 43.1%, respectively (*p* < 0.05). This highlights the greater effectiveness of the transperineal method in detecting cancers in this difficult-to-access area. Given this marked difference in diagnostic yield, particularly in anatomically challenging anterior regions, we recommend that a transperineal biopsy be considered in patients with a prior negative transrectal biopsy, especially when preceded by multiparametric MRI to evaluate the anterior prostate.

The main objective of this study was to evaluate the detection of both clinically significant and insignificant prostate cancer, with a focus on refining biopsy techniques to enhance diagnostic precision and reduce the incidence of undetected lesions. This approach is essential for advancing the accuracy of prostate cancer detection and improving clinical decision-making.

The results of this study revealed a statistically significant increase in the detection of clinically insignificant prostate cancer in the transperineal biopsy group compared with the transrectal biopsy group (8.09% vs. 4.2%; *p* < 0.05). This underscores the superior diagnostic sensitivity of the transperineal approach, particularly in the detection of low-risk lesions, which may otherwise be missed or under-sampled with the transrectal biopsy technique. The enhanced sensitivity of the transperineal approach in detecting low-risk lesions, can improve diagnostic accuracy and minimize the risk of overdiagnosis in prostate cancer detection. The transperineal group also had a higher mean number of cores obtained per procedure (14.41 ± 2.60 vs. 13.96 ± 2.03), which may have contributed to the higher detection rate for low-risk lesions. The elevated sampling rate, combined with the integration of both systematic and targeted biopsy strategies, probably enhanced the diagnostic sensitivity, particularly for anterior and smaller-volume lesions.

Although the rate of clinically insignificant prostate cancer detected in our transperineal cohort was slightly higher than typically reported in the literature, the approach concurrently demonstrated improved detection of clinically significant cancers, reinforcing its diagnostic value. These results support the findings of Djavan et al., who observed a direct correlation between the number of biopsy cores and the detection rate of low-risk prostate cancer. This reinforces the significance of expanding the biopsy sampling process to improve diagnostic accuracy, particularly for low-risk lesions that might otherwise go undetected. This suggests that a higher volume of tissue sampling may improve the detection of clinically insignificant lesions. This underscores the importance of balancing diagnostic thoroughness with the risk of overdiagnosis [33].

In patients undergoing transrectal biopsy, prostate cancer detection rates were 31.1%, 83.2%, and 59.5% for the 50–60 years, 61–75 years, and over 75 years age groups, respectively. In comparison, transperineal biopsy demonstrated significantly higher detection rates in the 50–60-year age group, which includes patients most likely to benefit from curative treatment. Conversely, transrectal biopsy showed a higher detection rate primarily in the 61–75-year age group, suggesting its greater effectiveness in this older cohort. A multicenter study in the literature [34] reports a prostate cancer detection rate of 30.5% for patients aged over 75 years, compared to 5.2% for those under 50 years. However, the majority of these diagnoses are clinically insignificant prostate cancers. In contrast, our study revealed a significantly higher detection rate in the 50–60-year age group using the transperineal biopsy approach (62.1% vs. 31.1%), exceeding the detection rates documented in existing studies. Prostate cancer detection rates were significantly higher with transperineal (TP) biopsy compared with transrectal (TR) biopsy across all PSA ranges (*p* < 0.05). In patients with PSA levels < 10 ng/mL, detection was 54% with TP biopsy versus 37% with TR. For PSA levels between 10.01 and 20 ng/mL, detection rates were 59% for TP and 47% for TR. In patients with PSA > 20 ng/mL, detection rates were 84% for TP and 75% for TR. The greatest difference was seen in the PSA < 10 ng/mL group, suggesting that TP biopsy is more effective in early-stage or lower-PSA cases (*p* < 0.05).

These findings support previous studies that emphasize the advantages of the transperineal biopsy technique, particularly for patients with PSA levels of 4.01–10 ng/mL, where clinically significant cancers are frequently missed by the transrectal approach. The transperineal method improves the detection of such cancers in this range [35,36]. These findings suggest that the transperineal approach enhances the detection of prostate cancer at a localized stage, probably due to improved access to anatomically challenging regions such as the anterior and apical zones. Within the PSA range of 4–10 ng/mL, previous studies have reported prostate cancer detection rates using systematic biopsy techniques ranging from 30% to 50%. The higher detection rates observed in our study using the transperineal technique suggest a potential diagnostic advantage in this clinically uncertain PSA interval [37,38,39,40]. In contrast, our study reported a detection rate of 54% within this PSA range (4–10 ng/mL), exceeding previously published figures and suggesting a potentially superior diagnostic performance of the transperineal approach in this intermediate-risk cohort.

Strengths of this Study:

The large size of the patient cohort (*n* = 1674) enhances the reliability and generalizability of the results. A major advantage is the direct comparison between transperineal and transrectal prostate biopsy techniques, both performed under local anesthesia and in similar clinical conditions. The focus on clinically significant prostate cancer, as well as the superior detection of anteriorly located tumors using the transperineal approach, increases the clinical relevance of the findings. Moreover, the combined use of systematic and MRI-targeted biopsies reflects current best practices in prostate cancer diagnosis.

Limitations of this Study:

This study’s retrospective design may have introduced selection bias and limits the ability to control for confounding factors. Pain perception was not evaluated using a standardized scoring system, which restricts the assessment of patient comfort across techniques. Furthermore, while all transperineal biopsies were performed by a single surgeon, the transrectal procedures were carried out by multiple operators, potentially introducing variability in technique and outcomes. This study does not include long-term follow-up data, such as treatment decisions or patient survival.

## 5. Conclusions

This study evaluates the diagnostic efficacy of cognitively guided, freehand transperineal prostate biopsy performed through a single perineal access point, in comparison to the conventional transrectal biopsy technique. The primary objective is to determine whether the transperineal approach offers a higher overall prostate cancer detection rate, with particular emphasis on its ability to improve sampling of the anterior prostate zone, which is frequently under-sampled or missed altogether by the transrectal route due to anatomical limitations. All transperineal prostate biopsies were conducted by a single urologic surgeon, who attained procedural proficiency following the completion of approximately 40 cases, aligning with established benchmarks for the transperineal biopsy learning curve reported in the literature. This approach ensured procedural consistency and minimized operator-dependent variability.

In addition to performing systematic biopsy involving the retrieval of 12 cores, by using a cognitive targeting method based on previous imaging, this approach offers a simpler and potentially more accurate way to locate and sample suspicious areas. This study included a large group of patients who met specific inclusion criteria, enabling meaningful comparisons. The findings may help improve diagnostic accuracy, reduce the risk of infection, and support wider use of the transperineal technique in everyday clinical practice. The ultrasound-guided transperineal technique also allows for improved characterization of suspicious lesions visualized on ultrasound, enabling precise needle placement and facilitating targeted biopsies of identified areas.

A distinctive feature of this study, aside from the large patient cohort, was the use of a minimally invasive transperineal biopsy technique performed entirely under local anesthesia. The procedure involved a single, small perineal skin incision, through which targeted tissue samples were obtained from both lobes of the prostate using a cognitively guided, freehand approach. This method eliminated the need for multiple punctures or advanced fusion systems, while still ensuring adequate and anatomically comprehensive sampling. The technique proved to be both efficient and well tolerated by patients, further supporting its feasibility in routine outpatient settings.

## Figures and Tables

**Table 1:** Demographic and clinical characteristics of the study cohort and distribution of covariates and statistical tests between matched groups.

	Group I Transperineal (1161)	After PSM Group I Transperineal (513)	Group II Transrectal (513)	After PSM Group II Transrectal (513)	*p*-Value	After PSM *p*-Value
Age (mean ± standard deviation, median)	69.87 ± 7.71	66.91 ± 7.24	72.19 ± 7.81	72.19 ± 7.82	<0.001 (Student)	<0.001 (Mann–Whitney U)
	70.00	67.00	72.00	72.00		
PSA (mean ± standard deviation, median)	53.8437 ± 128.29	40.71 ± 108.75	72.7547 ± 162.23	72.75 ± 162.24	<0.001 (Mann-Whitney)	<0.001 (Mann-Whitney U)
	15.3000	13.00	19.9800	19.98		
Rectal digital exam (yes/no)	587/574	191/322	317/196	317/196	<0.001 (Chi-square)	<0.001 (Chi-square)
Prostate volume (mean ± standard deviation, median)	64.43 ± 29.76	64.35 ± 30.24	64.34 ± 29.20	64.34 ± 29.21	0.954 (Student)	0.919 (Mann–Whitney U)
	59.00	58.00	57.00	57.00		
I No. of cores (mean ± standard deviation, median)	14.41 ± 2.60	15.04 ± 3.21	13.96 ± 2.03	13.96 ± 2.04	<0.001 (Student)	<0.001 (Mann–Whitney U)
	14.00	14.00	14.00	14.00		
Obesity (yes/no)	603/558	274/239	265/248	265/248	0.915 (Chi-square)	0.617 (Chi-square)
Anticoagulant (yes/no)	407/754	208/305	151/362	151/362	0.024 (Chi-square)	0.0002 (Chi-square)
Diabetes (yes/no)	394/767	236/277	136/377	136/377	0.003 (Chi-square)	<0.001 (Chi-square)
MRI (yes/no)	644/517	297/216	252/261	252/261	0.016 (Chi-square)	0.0059 (Chi-square)
US with elastography (yes/no)	453/708	315/198	127/386	127/386	<0.001 (Chi-square)	<0.001 (Chi-square)
Prior biopsies (yes/no)	188/973	166/347	33/480	33/480	<0.001 (Chi-square)	<0.001 (Chi-square)

PSA = prostate-specific antigen, MRI = magnetic resonance imaging, US = ultrasound.

**Table 2:** Characteristics of patients diagnosed or not with prostate cancer.

	No Prostate Cancer (*n* = 581)			Prostate Cancer (*n* = 1093)			
	Mean	Median	Range	Mean	Median	Range	*p*-Value *
Age	70.27 7.51	70	40–91	70.74 7.97	70	46–93	>0.05
PSA	19.94 46.52	11.48	1.16–1000	80.73 165.85	23	0.62–1000	<0.05
Transperineal biopsies (number)	373			788			
Transrectal biopsies (number)	208			305			
Prostate volume	65.80 29.88	60	16–187	63.67 29.40	58	14–229	>0.05

PSA = prostate-specific antigen. * Indicates the statistically significant *p*-value obtained from the Mann–Whitney U test.

**Table 3:** Overall Efficacy of Prostate Cancer Detection.

	TPPBx *n* = 1161	TRPBx *n* = 513	*p* Value
Detection of clinically insignificant prostate cancer iPCa (Gleason = 6)	94 (8.1%)	22 (4.3%)	*p* > 0.05
Detection of clinically significant prostate cancer csPCa (Gleason ≥ 7, GG ≥ 2)	669 (57.6%)	272 (53%)	*p* > 0.05
No prostate cancer	373 (32.1%)	208 (40.5%)	*p* > 0.05
Total detection of prostate cancer (all histological forms)	788 (67.8%)	305 (59.4%)	*p* > 0.05
Prostate cancer detection (Gleason score)	763 (65.7%)	294 (57.3%)	*p* < 0.05

**Table 4:** Gleason Score and Prostate Cancer Detection.

		Procedure		Total	*p* Value
		TPPBx	TRPBx		
No prostate cancer		398	219	617	
		34.3%	42.7%	36.9%	>0.05
6 (3-3)	Number (n)	94	22	116	
	Percent %	8.1%	4.3%	6.9%	<0.05
7 (3-4)	Number (n)	77	32	109	
	Percent %	6.6%	6.2%	6.5%	>0.05
7 (4-3)	Number (n)	157	53	210	
	Percent %	13.5%	10.3%	12.5%	>0.05
8 (4-4)	Number (n)	262	96	357	
	Percent %	22.5%	18.7%	21.3%	>0.05
9 (4-5)	Number (n)	113	49	162	
	Percent %	9.7%	9.6%	9.7%	>0.05
9 (5-4)	Number (n)	24	14	38	
	Percent %	2.1%	2.7%	2.3%	>0.05
10 (5-5)	Number (n)	36	28	64	
	Percent %	3.1%	5.5%	3.8%	>0.05

**Table 5:** Risk Group Stratification of Prostate Cancer Patients Based on Age.

	TPPBx			TRPBx			*p* Value	*p* Value	*p* Value
		Age Categories (Years)			Age Categories (Years)				
Risk Groups	50–60 y (*n*) = 95	61–75 y (*n*) = 777	>75 y (*n*) = 283	50–60 y (*n*) = 39	61–75 y (*n*) = 294	>75 y (*n*) = 178	TP vs. TR	TP vs. TR	TP vs. TR
Low	11 (11.6%)	102 (13.1%)	23 (8.1%)	5 (12.8%)	26 (8.8%)	12 (6.7%)		*p* > 0.05	
Intermediate	26 (27.4%)	205 (26.4%)	86 (30.4%)	9 (23.1%)	57 (19.4%)	32 (18%)		*p* > 0.05	*p* < 0.05
High	22 (23.2%)	187 (24.1%)	99 (35%)	12 (30.8%)	77 (26.2%)	62 (34.8%)		*p* > 0.05	*p* > 0.05

TPPBx = transperineal prostate biopsy, TRPBx = transrectal prostate biopsy TP = transperineal route, TR = transrectal route.

**Table 6:** Risk Groups According to PSA.

	PSA < 10 ng/mL		PSA 10–20 ng/mL		PSA > 20 ng/mL	
	TP	TR	TP	TRP	TPP	TR
Prostate cancer detection	201/368 (54%)	40/106 (37%)	190/322 (59%)	72/151 (47%)	397/471 (84%)	193/256 (75%)
*p* value	<0.05		<0.05		<0.05	
GG ≥ 2	143/368 (38%)	29/106 (27%)	157/322 (48%)	55/151 (36%)	369/471 (78%)	188/256 (73%)
*p* value	<0.05		<0.05		> 0.05	
GG ≥ 3	124/368 (33%)	27/106 (25%)	140/322 (43%)	43/151 (28%)	328/471 (69%)	170/256 (66%)
*p* value	>0.05		<0.05		>0.05	

GG = grade group, TP = transperineal route, TR = transrectal group, PSA = prostate-specific antigen. The intermediate-risk group consists of two subgroups: GG ≥ 2—Gleason score 7 (3 + 4) and GG ≥ 3—Gleason score 7 (4 + 3). The high and very high-risk group includes two subgroups: GG4—Gleason score 8 and GG5—Gleason score 9–10.

## Data Availability

We do not provide additional data.

## Abbreviations

The following abbreviations are used in this manuscript: TPPBx—transperineal ultrasound-guided prostate biopsy, TRPBx—transrectal ultrasound-guided prostate biopsy, PSA—prostate-specific antigen, MRI—magnetic resonance imaging, GG—grade group, TP—transperineal route, TR-transrectal route, csPCa—clinically significant prostate cancer, iPCa—clinically insignificant prostate cancer, US—ultrasound, PSM—propensity score matching.
